# Cost-effectiveness of the prevention of adhesions and adhesive small bowel obstruction after colorectal surgery with adhesion barriers: a modelling study

**DOI:** 10.1186/s13017-019-0261-2

**Published:** 2019-08-16

**Authors:** Pepijn Krielen, Janneke P. C. Grutters, Chema Strik, Richard P. G. ten Broek, Harry van Goor, Martijn W. J. Stommel

**Affiliations:** 1 Department of Surgery, Radboud University Medical Center, Nijmegen, The Netherlands; 2 Department for Health Evidence, Radboud University Medical Center, Nijmegen, The Netherlands; 3 Department of Operating Rooms, Radboud University Medical Center, Nijmegen, The Netherlands

**Keywords:** Adhesions, Adhesion barrier, Colorectal surgery, Cost-effectiveness

## Abstract

**Background:**

Adhesion barriers have proven to reduce adhesion-related complications in colorectal surgery. However, barriers are seldom applied. The aim of this study was to determine the cost-effectiveness of adhesion barriers in colorectal surgery.

**Methods:**

A decision-tree model was developed to compare cost-effectiveness of no adhesion barrier with the use of an adhesion barrier in open and laparoscopic surgery. Outcomes were incidence of clinical consequences of adhesions, direct healthcare costs, and incremental cost-effectiveness ratio per adhesion prevented. Deterministic and probabilistic sensitivity analyses were performed.

**Results:**

Adhesion barriers reduce adhesion incidence and incidence of adhesive small bowel obstruction in open and laparoscopic surgery. Adhesion barriers in open surgery reduce costs compared to no adhesion barrier ($4376 versus $4482). Using an adhesion barrier in laparoscopic procedures increases costs by $162 ($4482 versus $4320). The ICER in the laparoscopic cohort was $123. Probabilistic sensitivity analysis showed 66% and 41% probabilities of an adhesion barrier reducing costs for open and laparoscopic colorectal surgery, respectively.

**Conclusion:**

The use of adhesion barriers in open colorectal surgery is cost-effective in preventing adhesion-related problems. In laparoscopic colorectal surgery, an adhesion barrier is effective at low costs.

## Introduction

Colorectal surgery commonly induces post-operative adhesion formation, causing a lifelong risk for small bowel obstruction, female infertility, and chronic visceral pain [1–4]. Lysis of adhesions at reoperative surgery is associated with inadvertent organ injury, prolonged operative time, and an increased risk of post-operative complications and, therefore, higher costs [5–7]. Several types of adhesion barriers are developed to prevent post-operative adhesion formation after abdominal surgery. In a recent systematic review and meta-analysis on efficacy and safety of adhesion barriers, hyaluronate carboxymethylcellulose (HA/CMC) was proven to safely reduce the incidence of site-specific adhesions and the incidence of re-operations for adhesive small bowel obstruction after open colorectal surgery [8]. However, despite the burden of post-operative adhesions, and the proven benefit of adhesion barriers, they are seldom applied. In a nationwide survey carried out in the Netherlands in 2009, just 13.4% of surgeons indicated that they had used any adhesion barrier in the previous year and a recent follow-up survey did not report much subsequent change [9, 10]. Doubts about cost-effectiveness and the need for adhesion prevention in minimally invasive surgery may explain the reluctance in the use of barriers. Previous cost-effectiveness analyses of adhesion barriers have been based on costs of adhesion-related re-admission and only concern open surgery [11, 12]. The efficacy data used were derived from second-look surgery studies, with a suggested 25–50% reduction in the number or density of adhesions with the use of a barrier [11, 12]. In the absence of data on reduction of adhesion-related readmissions with the use of a barrier, costs were extrapolated from the reduction of adhesions. Since publication of these analyses, evidence on both the burden of adhesions and the effectiveness of adhesion barriers has increased substantially. Earlier, re-admission for post-operative small bowel obstruction was considered the most important complication [13]. New evidence has clearly shown that difficulty due to dissecting adhesions at repeat abdominal surgery is an even bigger problem [14]. Moreover, evidence on efficacy of adhesion barriers is no longer limited to adhesion incidence, but comprises clinically relevant endpoints [8].

A decision-tree model was developed in this study for the use of an adhesion barrier in open colorectal surgery and laparoscopic colorectal surgery, based on the best available evidence and considering cost and effect. The model was designed as an important contribution towards creating an evidence-based, decision-making protocol on the use of adhesion barriers in colorectal surgery.

## Materials and methods

### Decision model

**Fig. 1:**
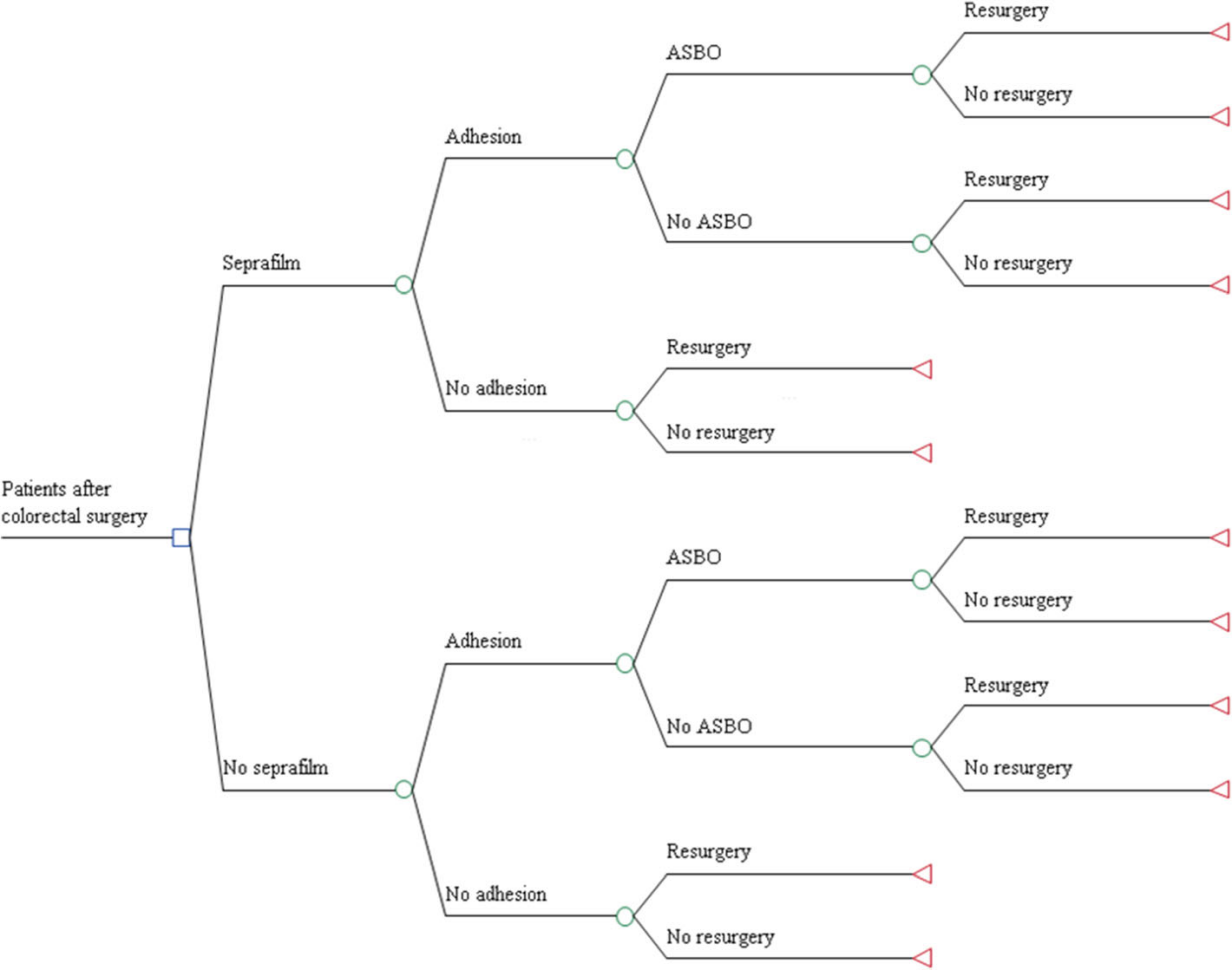
Decision-tree model for evaluation of the use of an adhesion barrier in colorectal surgery

A decision-tree model was designed with Microsoft Office Excel 2007 that evaluated the strategy of adhesion prevention with an adhesion barrier in both open and laparoscopic colorectal surgery. A decision-tree model is a simplified framework of complex real-life processes that uses a mathematical method to weigh the risks, benefits, and costs of clinical strategies [15]. In the model, two strategies are compared: (1) current clinical practice, colorectal surgery without the use of an adhesion barrier, and (2) colorectal surgery with the use of an adhesion barrier (Fig. 1).

Hypothetical cohorts of patients, who have undergone colorectal surgery (open or laparoscopic), were distributed over the different pathways in the decision-tree, based on a set of probabilities that were derived from recently published systematic reviews and observational and intervention studies. This allowed the synthesis of evidence and, thereby, evaluation of the effects and adhesion-related healthcare costs determined by the treatment decision.

Adhesive small bowel obstruction (ASBO) and difficulties at reoperation were included in the model as potential consequences of adhesions. Female infertility and chronic visceral pain were not considered. Risk of infertility is only an additional reason for the use of an adhesion barrier in a very small and specific subgroup. Regarding chronic visceral pain, no consistent evidence is available on etiology, incidence, and costs after colorectal surgery [4].

### Population

The two target populations consist of patients who undergo a colorectal resection for a benign or malignant indication, by either an open or laparoscopic surgical approach. Colorectal resection is commonly performed for various indications; the main indication is colorectal cancer [16]. Colorectal surgery has a relatively high incidence of postoperative adhesion formation [14, 17]. In 2016, in more than 85% of colorectal cancer resections performed in the Netherlands, laparoscopic techniques were used [18]. There is recent evidence that laparoscopy is associated with a lower incidence of adhesions, particularly to the abdominal wall [19, 20].

### Probabilities

In the model, the hypothetical cohorts of patients, who underwent a colorectal resection, with or without the use of an adhesion barrier, have different probabilities for the development of adhesions and subsequent development of ASBO, operative or conservative treatment for ASBO, and adhesiolysis at future repeat surgery. Probability estimates were derived from recent literature (see Table 1) [8, 19, 21–32]. PubMed, Embase, and the Cochrane Library were searched for relevant literature. Risk ratios for adhesions, ASBO, and operative treatment of ASBO, with the use of an adhesion barrier, are based on efficacy data for HA/CMC, as this is the only form of adhesion barrier with consistent evidence available on adhesion prevention in visceral surgery. HA/CMC is not easily applicable in laparoscopic surgery, and evidence for laparoscopy is lacking. Since there are no alternative barriers with sound evidence on safety and efficacy in laparoscopic colorectal surgery, efficacy data of HA/CMC in open colorectal resection was extrapolated to the laparoscopic model. The data on incidence of adhesions after open and laparoscopic colorectal surgery were derived from a recently published multicentre study [19]. In this study, adhesions after open and laparoscopic colorectal cancer surgery are compared during surgery for liver metastases.

**Table 1:** Input probabilities in decision-tree model

	Open			Laparoscopic				Adhesion barrier strategy		
Variable	Probability	*α*	*β*	Probability	*α*	*β*	Source (reference)	RR	95% CI	Source (reference)
Patients with adhesions	0.889	80	10	0.623	38	23	[19]	0.51	0.43–0.61	[8, 20–22]
Patients with ASBO 4 years	0.0856	199	2127	0.0663	77	1085	[23–30]	0.68	0.35–1.32	[8, 20–22]
Patients with ASBO treated surgically	0.032	74	2252	0.031	36	1126	[23–30]	0.49	0.28–0.88	[8, 20–22]
Patients with repeat surgery 4 years	0.208	64	200	0.209	64	200	[31]			

*α* patients with event, *β* patients without event, *RR* relative risk, *CI* confidence interval, *ASBO* adhesive small bowel obstruction

In a recent systematic review on the value of adhesion barriers, there were no data on the total incidence of adhesions with the use of HA/CMC [8]. A new search yielded no additional data on the total incidence of adhesions with the use of HA/CMC. Thus, adhesion incidence with HA/CMC was derived from the incidence of site-specific adhesions reported (i.e. midline, pelvic adhesions), by only including the anatomical site with the highest incidence of adhesions from each study [21–23]. The peristomal site was not considered relevant for total adhesion formation after colorectal surgery. The efficacy is expressed as a risk ratio of adhesions with the use of HA/CMC versus no adhesion barrier (RR 0.51 [95% CI 0.43–0.61]).

The probability of ASBO and the probability of surgery for ASBO after colorectal surgery were derived from an update of the systematic review on the burden of adhesions after abdominal surgery (1990 to June 2016) [24–31]. Weighted mean follow-up of the studies was 55.3 months. The probability of future repeat abdominal surgery was derived from a recently published, prospective cohort of patients, who underwent elective abdominal surgery [32]. This cohort comprises mainly patients operated by open approach. Since the incidence of repeat abdominal surgery is not expected to be substantially different for patients operated on by laparoscopy or by open approach, the probability used in both arms of the model is based on the total cohort. In the 4 years following initial lower gastrointestinal tract surgery, 24% of patients underwent repeat abdominal surgery, including re-operations for ASBO. In the model, re-operations for ASBO were subtracted from the probability for repeat surgery to ensure that these re-operations were not included twice in the model.

### Costs

An analysis of adhesion-related costs was performed with a healthcare perspective, including only direct healthcare costs for treatment (Table 2). All monetary values are presented in US dollars (USD/$). Euros were converted to USD using the exchange rate: 1 Euro to 1.1264 USD.

**Table 2:** Costs used in the model

	Value	SD	Source (reference)
Costs HA/CMC	$630	$382–$763*	[20, 32]
ASBO with operative treatment	$18366	$2831	[33]
ASBO with non-operative treatment	$2565	$299	[33]
Repeat surgery—no adhesions	$14063	$812	[5]
Repeat surgery—adhesions	$18579	$1722	[5]

*HA/CMC* hyaluronate carboxymethylcellulose, *ASBO* adhesive small bowel obstruction, *SD* standard deviation

*For the number of sheets per patient, a Beta-PERT distribution was assigned, ranging between 2 and 4

The mean number of films per patient reported in two of the three studies on adhesion prevention with HA/CMC in colorectal surgery was 3.3 films. The total costs for HA/CMC were based on the use of 3.3 films and the price of a HA/CMC film in 2016 in the Netherlands, adding up to a total cost of $629.68 [21, 33]. For sensitivity analysis, a Beta-PERT distribution was assigned for the number of sheets per patient, ranging between 2 and 4. Costs of the barrier were varied according to the Beta-PERT distribution ($382–$763), Table 2.

The healthcare costs of ASBO were derived from a recently performed retrospective analysis of patients admitted to the Radboud University Medical Center with the diagnosis of ASBO [34]. The costs for repeat surgery were derived from a recent, large, cohort study on adhesiolysis-related morbidity in abdominal surgery [5].

### Data analysis

Data were analysed using mean values for a base case analysis, to obtain percentages of ASBO, re-operation for ASBO, patients with adhesions, and direct healthcare costs for the two strategies, in the 4 years following colorectal surgery. The time frame was based on the mean 4 years’ follow-up periods of the studies, which underlie the probabilities for ASBO and repeat surgery. If the use of an adhesion barrier was more effective and more expensive, incremental cost-effectiveness ratios (ICER) were calculated to determine the additional costs for one patient, in whom adhesion formation was prevented. All presented ICERs are a comparison of the adhesion barrier strategy versus no barrier. If the adhesion barrier strategy was more effective and reduced costs, this was considered dominant, and ICERs were not calculated. A base case analysis was conducted for the two strategies in open and laparoscopic surgery separately.

Probabilistic sensitivity analysis was performed, using a Monte Carlo simulation, to explore the impact of uncertainties in the model parameters, as shown in Tables 1 and 2. In the Monte Carlo simulation, 5000 samples were drawn from the parameter distributions. For each sample, the hypothetical patient cohort was run through the model based on these sampled parameters, representing the uncertainty in the cost-effectiveness estimation. Lognormal distributions were used for all risk ratios; beta distributions for probabilities and costs were described by normal distributions. Confidence intervals were calculated from the probabilistic sensitivity analysis using the percentile method.

In addition, threshold analyses were conducted for the costs of the adhesion barrier and the probability of repeat surgery, in order to find the maximum values for these parameters at which the adhesion barrier saves costs. Deterministic sensitivity analysis was conducted to explore the influence of deviation in the efficacy of the different parameters on the cost-effectiveness, assuming all other variables to be fixed. All parameters were individually changed to their lower and upper boundaries of the 95% confidence intervals. Results of the analysis are presented in a tornado diagram. Furthermore, a best- and worst-case scenario was calculated; for the worst-case scenario, the risk ratios for adhesions, ASBO, and operative treatment of ASBO were all set to the upper limit of their confidence interval (Table 1). For the best-case scenario, all three risk ratios were raised to the lower limit of their confidence interval.

## Results

### Base case analysis

With the parameters at their base case values, for the open colorectal surgery cohort, the adhesion barrier strategy was both more effective and less expensive than the no adhesion barrier strategy, whilst in the laparoscopic colorectal surgery cohort, the adhesion barrier strategy was more effective, but more expensive (Table 3). In open colorectal surgery, use of an adhesion barrier reduced the incidence of adhesions from 88.9% (95% CI 81.8–94.5%) to 45.3% (95% CI 37.3–54.6%) and the incidence of ASBO from 8.6% (95% CI 7.5–9.7%) to 6.2% (95% CI 2.9–11.3%). The expected mean direct healthcare costs in 4 years after initial open colorectal surgery were reduced by $106, from $4482 (95% CI $3074–$6284) per patient in the group without an adhesion barrier to $4376 (95% CI $3140–$5892) in the group with an adhesion barrier. After laparoscopic colorectal surgery, the incidence of patients with adhesions was reduced from 62.3% (95% CI 49.9–73.8%) to 31.8% (95% CI 24.3–40.7%) and the incidence of ASBO from 6.6% (95% CI 5.2–8.1% ) to 4.5% (95% CI 2.2–9.2%) with an adhesion barrier. Costs increased by $163 per patient when an adhesion barrier was used. Direct health care costs over 4 years after laparoscopic colorectal surgery for the adhesion barrier group were $4482 (95% CI $3031–$5591) versus $4320 (95% CI $2881–$5 709) for the no adhesion barrier group.

**Table 3:** Results of base case and deterministic sensitivity analyses in the open and laparoscopic surgery cohorts

Strategy	Costs	Percentage adhesions	Percentage ASBO	Costs per patient with adhesions prevented
Open cohort				
Baseline				
No barrier	$4474	88.9%	8.6%	
Barrier	$4372	45.3%	5.8%	Dominant
Best-case scenario				
No barrier	$4474	88.9%	8.6%	
Barrier	$4129	38.2%	3.0%	Dominant
Worst-case scenario				
No barrier	$4474	88.9%	8.6%	
Barrier	$4789	54.2%	11.3%	$908
Laparoscopic cohort				
Baseline				
No barrier	$4179	62.3%	6.6%	
Barrier	$4220	31.8%	4.5%	$135
Best-case scenario				
No barrier	$4179	62.3%	6.6%	
Barrier	$4016	26.8%	2.3%	Dominant
Worst-case scenario				
No barrier	$4179	62.3%	6.6%	
Barrier	$4576	38.0%	8.7%	$1663

Cost reduction for both open and laparoscopic colorectal surgery is mainly due to the reduction of readmissions for ASBO in the adhesion barrier arm. Reduction of costs is also due to the prevention of adhesions at reoperation and therefore reduction of operative time with a decrease of time needed for adhesiolysis.

In open colorectal surgery, the adhesion barrier strategy dominated the current, no-adhesion barrier practice. For laparoscopic colorectal surgery, the ICER for one patient with adhesions prevented was $123.

### Sensitivity analysis

**Fig. 2:**
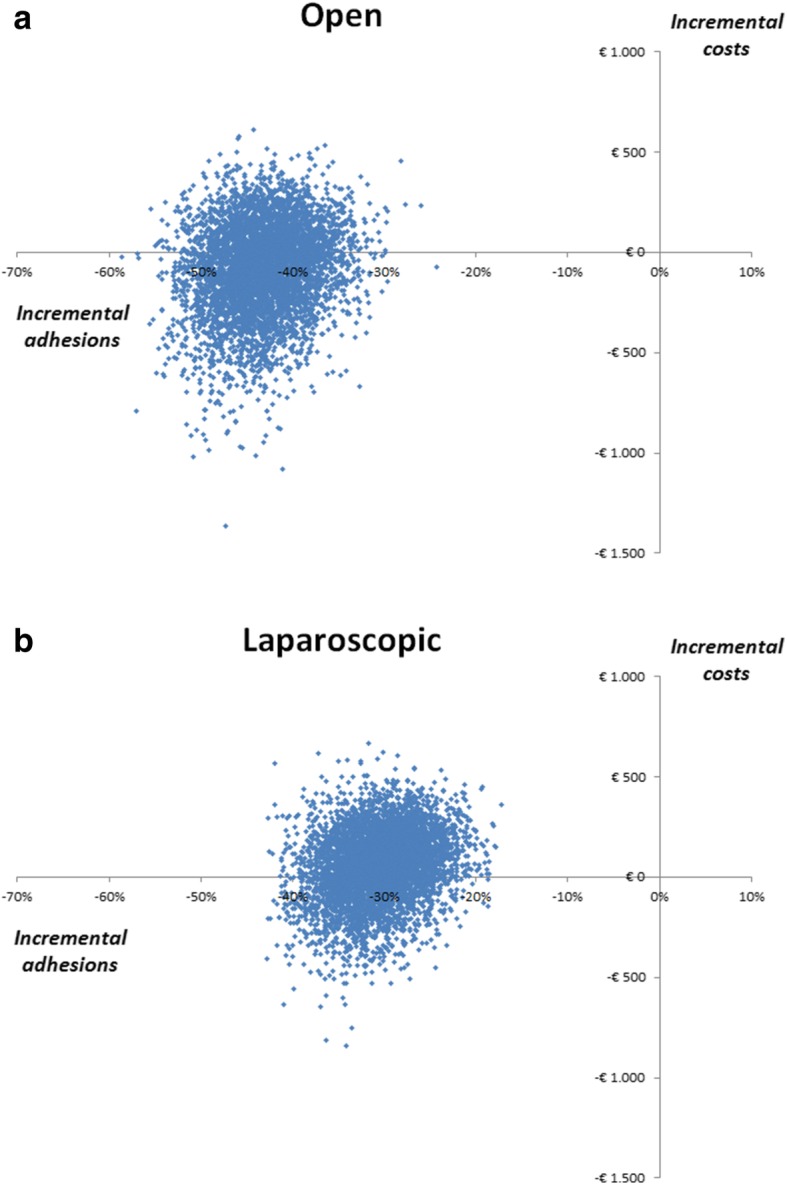
**a** Scatter plot of Monte Carlo simulation for open colorectal surgery, displaying cost (*y*-axis) and effect (*x*-axis) of adhesion barrier strategy. **b** Scatter plot of Monte Carlo simulation for laparoscopic colorectal surgery, displaying cost (*y*-axis) and effect (*x*-axis) of adhesion barrier strategy

The results of the probabilistic sensitivity analysis are shown in Fig. 2a and b. The Monte Carlo simulation showed that the use of an adhesion barrier is always more effective in preventing adhesions and ASBO, for both open and laparoscopic colorectal surgery. The use of an adhesion barrier had a 66% probability of reducing costs in the open surgery cohort. In the laparoscopic surgery cohort, the probability was 41%.

Threshold analysis in the open colorectal surgery cohort showed that using a barrier priced at $736 (95% CI $305–$1187) or more no longer reduces costs. The same effect was seen with the re-operation rate lowered to 16% (95% CI 1 - 74%) or less. In the laparoscopic surgery cohort, the thresholds for cost-reduction with an adhesion barrier were a price of $592 (95% CI $256–$954) and a re-operation rate of 24% (95% CI 3–100%).

**Fig. 3:**
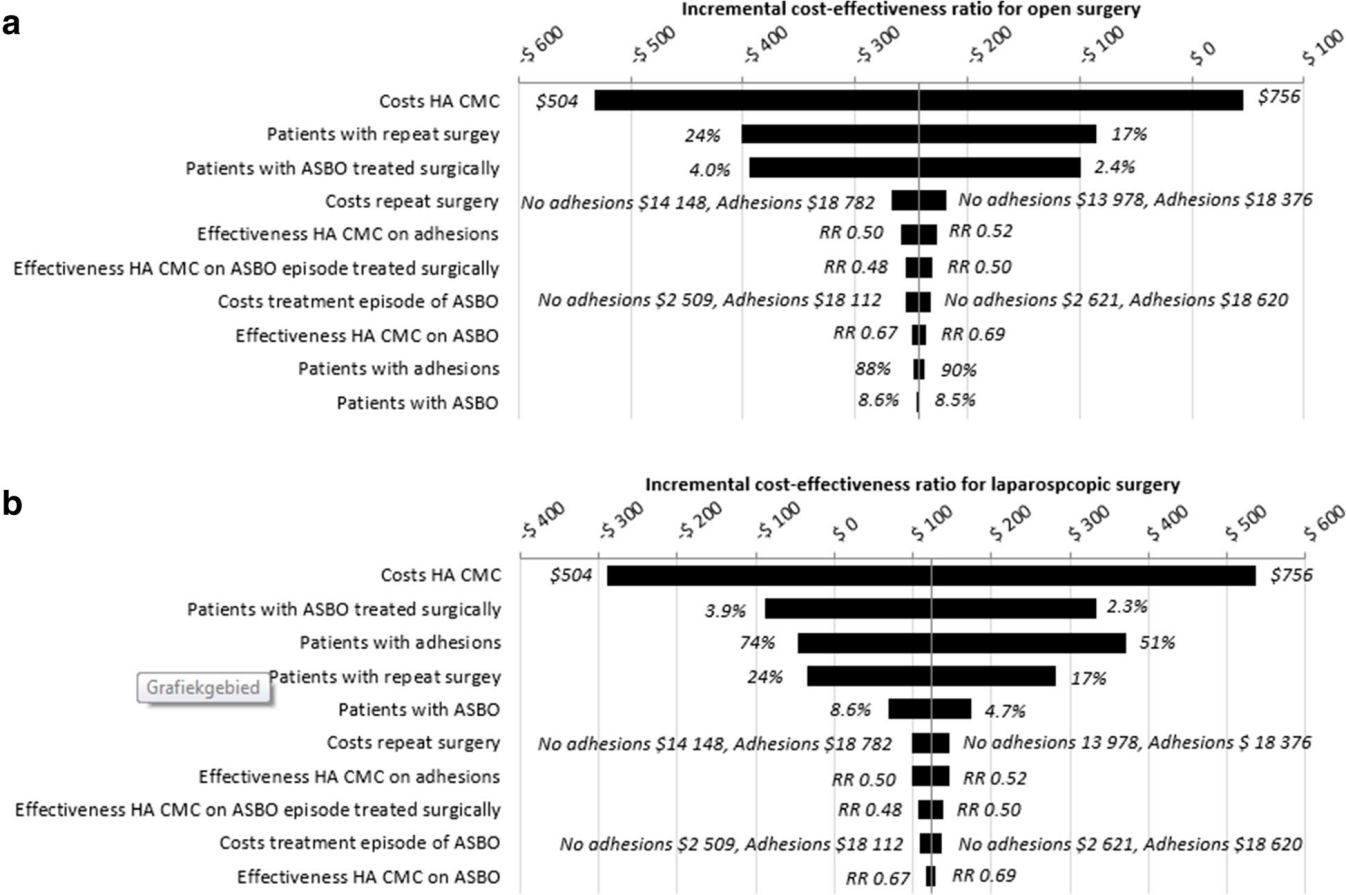
**a** Tornado diagram of variation of individual parameters in open colorectal surgery. **b** Tornado diagram of variation of individual parameters in laparoscopic colorectal surgery

Results of the deterministic sensitivity analysis are shown in Fig. 3a and b. Variation of the costs of the adhesion barrier had the largest effect on the ICER for one patient with adhesions prevented in open and laparoscopic surgery. In the best-case scenario, applying an adhesion barrier in both open and laparoscopic colorectal surgery reduces costs. In the worst-case scenario, the ICER for one patient with adhesions prevented is $908 in the open colorectal surgery patient cohort and $1663 in the laparoscopic colorectal surgery patient cohort, Table 3.

## Discussion

The routine use of an adhesion barrier in open colorectal surgery is cost-effective, considering a 4-year time frame. Whilst in laparoscopic colorectal surgery, the expenses are only $163 per patient, and the additional costs for one patient with adhesions prevented are $123.

The findings in the present study are in agreement with a comparable study, which demonstrated cost savings in all types of open abdominal surgery and potential cost-effectiveness in major laparoscopy [35]. The present study has the advantage that it concerns a homogenous group of patients with a high risk of post-operative adhesion formation. This well-defined population enhances the clinical applicability of the results. In addition, more recent cost data are used in the present model, of which the majority were specifically for colorectal surgery. Costs are twice as much for operative treatment of ASBO and for the adhesion barrier compared to costs reported previously. A comparable underestimation of costs for the adhesion barrier and ASBO treatment was found in other cost-effectiveness reports from earlier this century [11, 12]. The most important limitation of previous studies is the lack of evidence on efficacy of adhesion barriers in reducing adhesion-related complications.

The major strength of the present study is that the recently generated evidence for the burden of adhesions and the efficacy of adhesion barriers in colorectal surgery could be synthesized. We synthesized all available evidence to show the expected consequences of adopting adhesion barriers on both costs and effects, as well as the impact of the uncertainty due to a lack of evidence regarding these consequences. A limitation is the need to extrapolate data on the efficacy of adhesion barriers from open to laparoscopic colorectal surgery, due to scarce and inconsistent evidence with other formulas of HA/CMC (e.g. slurry made of film and spray) in laparoscopy [36–38]. A deviating efficacy in laparoscopy would be highly relevant, particularly, because the majority of colorectal resections are currently performed by laparoscopy [18]. In the worst-case scenario, assuming reduced effectiveness of the adhesion barrier (RR 0.61) resulted in an ICER of $908 in the open surgery cohort and $1633 in the laparoscopic surgery cohort, which for laparoscopy is more than a tenfold increase compared to base case analysis. Therefore, the modelled risk ratio (0.51) of adhesions with the use of an adhesion barrier should serve as reference standard for the development of novel adhesion barriers for laparoscopic use.

With the rise of laparoscopy in colorectal surgery, open surgery is almost exclusively performed in cases that are not suited for a laparoscopic approach. One of the reasons for an open approach could be problems with adhesions during laparoscopic surgery. Open cases are therefore more prone to postoperative complications [5]. This example illustrates the need for adhesion barriers in both laparoscopic and open surgery, to prevent future problems at repeat surgery.

The time frame, within which the model applies, was limited to 4 years, whilst adhesion-related complications or repeat surgery may occur many years later [13]. However, approximately 60% of ASBO occurs within the first 4 years after lower abdominal surgery [13]; there is no data available for repeat surgery. Using a longer time frame would increase ASBO and repeat surgery rate, thereby potentially increasing the clinical benefit and cost-effectiveness of the adhesion barrier strategy.

Female infertility and chronic visceral pain, which are known consequences of adhesions, were not included in the model. Risk for infertility is only applicable to a small group of female patients undergoing colorectal surgeries at a young age. No consistent evidence is available regarding chronic visceral pain, and most costs are generated outside the hospital [4]. The incompleteness of the model for these adhesion-related complications may have caused underestimation of adhesion-related costs, and thus an underestimation of the cost-effectiveness of the use of adhesion barriers.

The model took into account the costs of repeat surgery depending on the presence of adhesions, and not the extent and severity of adhesions. Evidence shows that laparoscopic approach and use of an adhesion barrier reduce the incidence of adhesions and their extent and severity [8, 19]. Although reduction of extent and severity of adhesions potentially decreases adhesiolysis-related complications and costs, the evidence was insufficient to consider including these variables in the model [5]. Excluding the efficacy and costs related to reduction in severity and extent may have resulted in an overestimation of the adhesion-related costs in the laparoscopic surgery cohort and an underestimation of the benefit of an adhesion barrier in both cohorts.

The costs of an adhesion barrier were based on the unit costs in the Netherlands in 2016. The unit costs may change according to the volume of products required. Variation of the costs of an adhesion barrier had the largest influence in our model, Fig. 3a and b*.* Higher volumes may result in a lower unit cost, favouring the cost-effectiveness of the use of adhesion barriers in colorectal surgery.

Due to a higher life expectancy and advances in surgical technology, an increasing number of patients undergo abdominal surgery multiple times during their lifetime [32]. Adhesion formation is the most common long-term complication of abdominal surgery, and prevention of adhesion formation from initial abdominal surgery is the critical step in breaking the sequence of complications due to adhesions. Despite evidence of reduced adhesion formation with the application of adhesion barriers, adhesion barriers are seldom used in practice. Doubts about cost-effectiveness and the need for adhesion prevention in the ‘minimally invasive era’ probably underlie this reluctance [9]. The present cost-effectiveness analysis is based on the best evidence available for both open and laparoscopic colorectal surgery and can, for open colorectal surgery at least, remove these doubts. Since the use of an adhesion barrier in laparoscopic colorectal surgery involves extra costs, data on quality-adjusted life-years (QALYs) are required to value the benefits of adhesion barriers and to compare the costs per unit of effect gained to a cost-effectiveness threshold [39]. In order to determine QALYs for adhesions and the use of adhesion barriers, future research should address patient-reported outcomes (PROs), such as functional status and quality of life. It is conceivable that adhesion-related complications will have a negative impact on PROs [40].

## Conclusion

The use of an adhesion barrier in open colorectal surgery will probably result in cost savings, and in laparoscopic colorectal surgery, this might be accompanied by limited additional costs. For laparoscopic colorectal surgery, more evidence on adhesion barriers is a prerequisite for clinical implementation.

## Data Availability

The datasets used and/or analysed during the current study are available from the corresponding author on reasonable request.

## Abbreviations

ASBO: Adhesive small bowel obstruction
CI: Confidence interval
HA/CMC: Hyaluronate carboxymethylcellulose
ICER: Incremental cost-effectiveness ratios
PROs: Patient reported outcomes
QALYs: Quality-adjusted life-years
RR: Risk ratio
USD: US dollars
